# Prevalence and correlates of antenatal depression at Chelstone First Level Hospital in Lusaka, Zambia: a cross-sectional study

**DOI:** 10.4314/ahs.v22i4.39

**Published:** 2022-12

**Authors:** Brian Maila, Ravi Paul, Sebean Mayimbo, Kelvin Kabwita

**Affiliations:** 1 School of Medicine, Department of Psychiatry, University of Zambia, P.O.Box 50110, Lusaka, Zambia; 2 Chainama Hills College Hospital, P.0.Box 30043, Lusaka, Zambia; 3 School of Nursing Sciences, University of Zambia, P.O.Box 50110, Lusaka, Zambia; 4 Lusaka District Health Office, Chelstone First Level Hospital, P.O. Box 310026, Lusaka, Zambia

**Keywords:** Antenatal depression, associated factors, depression in pregnancy, depressive symptoms, depression, pregnancy, antenatal care

## Abstract

**Background:**

Antenatal depression is associated with long-term disability in both mothers and new-borns. Inadequate data and research can constrain resource allocation and exacerbate the condition's symptoms.

**Objective:**

The purpose of this study was to determine the prevalence of prenatal depression and the characteristics associated with it among women receiving prenatal care at Chelstone First Level Hospital in Lusaka.

**Method:**

A cross-sectional survey of 281 pregnant women receiving prenatal care at Chelstone First Level Hospital was conducted using systematic random sampling. The Edinburgh Postnatal Depression Scale (EPDS) was used to assess participants' depression, and related data were collected using a structured, pretested, and interviewer-administered questionnaire.

**Results:**

Prenatal depression was identified in 26.3 percent of pregnant women surveyed (95 CI: 21% -32%), with antenatal depression being significantly more prevalent in women who did not have a satisfactory relationship with their partner/significant other (OR=1.70, 95CI: 1.40–3.10). Unemployment was found to be a risk factor for antenatal depression, with a 1.3 (95 CI:1.04–1.5) fold increased risk compared to employed women.

**Conclusion:**

Depressive symptoms are common among pregnant women seeking antenatal care in primary care, and unemployment, as well as a lack of relationship satisfaction with the spouse/significant other, increases the risk of depression.

## Introduction

Depression is a common psychiatric disorder and a major contributor to the global burden of disease affecting more than two hundred sixty-four million people globally of which the majority are women as compared to men^1^. The Diagnostic and Statistical Manual of Mental Disorders, 5^th^ edition (DSM-5) states that the twelve-month prevalence of major depressive disorder in the United States is roughly 7%, with females having 1.5- to 3-fold higher rates than males starting in early adolescence^2^.

Notwithstanding the variations in the prevalence rates of depression in pregnancy, studies conducted outside Africa agree that depression is the most common mental disorder in pregnancy. The reported prevalence rates have fluctuated from 6.9% in Malaysia^3^ to 81% in Hyderabad of Pakistan^4^. This variation can be explained by the differences in methodology, sample size, psychometric tools, cut-off point scores, differences in social-economic status and the differences in gestation age of the pregnancy at the time the studies were conducted.

According to The Harvard University Effective Altruism Student Group Philanthropy Advisory Fellowship 2015 Report, depression accounted for the largest burden of disease in Sub-Saharan Africa (SSA)^5^. Moreover, mental disorders and other non-communicable diseases are posing an increasing challenge for health systems in SSA, which have to date mostly focused on confronting infectious diseases, maternal, neonatal, and child deaths.^6^ The epidemiological transition has shifted the burden of mental and behavioural disorders to the adult population. 7 As a consequence of this shift, disorders such as Major Depressive Disorders are increasingly being seen in Sub-Saharan Adult population, especially women in the Child bearing age. For example, a cross-sectional survey of women attending antenatal clinic in Nigeria reported a prevalence of 24.5%^8^. Another cross-sectional study conducted among 393 pregnant women in public health centers of Ethiopia found a prevalence of 24.95% for antenatal depression^9^. Additionally, a study conducted at Gondar University Hospital in Northwest Ethiopia among 388 pregnant women was found a prevalence of 23%^10^. Furthermore, a systematic review and meta-analysis conducted in Ethiopia also found a high prevalence of antenatal depression ranging from 24.6%-27.1%^11^.

In Zambia, the magnitude of antenatal depression is not well understood. One cross-sectional study conducted at The University Teaching Hospital reported a prevalence of 42% out of 206 pregnant women interviewed^12^. A qualitative study conducted in the different stages of the perinatal period reported the presence of mental distress in the four different domains: worry about HIV status and testing; uncertainty about survival from childbirth; lack of social support; and vulnerability/oppression and concluded that identifying mental distress and prompt referral for interventions is critical to improving the mental health of the mother and prevent the effects of mental distress on the baby^13^.

Numerous studies conducted in various countries have identified the following risk factors for antenatal depression: young age^10^,^14^, single marital status, lower social economic status, and past negative obstetrical outcomes^15^, history of depression^11^,^16^, coexisting medical conditions like hypertension in pregnancy^8^,^12^, conflicts with the partner^17^, negative life events^18^ less education^19^, physical and psychological intimate partner violence^20^,^19^, drinking alcohol during pregnancy^8^, unplanned pregnancy and lack of intimate partner support^9^.

Prenatal psychiatric disorders are associated with suboptimal prenatal behaviour, including infrequent antenatal visits and exaggerated substance use disorders^21^,^22^. Therefore, allowing depression to continue into the postpartum period can have long-term consequences for both mother and baby, with mothers developing chronic mood disorders, and postpartum depression, which can impair mother-infant devotedness^23^. Moreover, being exposed to a chronically depressed mother can have cognitive, emotional, and behavioural aftermaths on the child^24^. Considering the adverse effects associated with antenatal depression, it is important that interventions to change health behaviours during pregnancy consider a woman's affective state, social context, and the overall mental wellbeing of pregnant women. Therefore, the present article highlights the prevalence of antenatal depression and the associated factors among pregnant mothers attending antenatal clinic (ANC) at Chelstone First Level Hospital in Lusaka.

## Materials and Methods

A cross sectional study was conducted among 1200 pregnant women attending antenatal clinic at Chelstone First Level Hospital, a site randomly selected from clinics offering antenatal care in a primary health care setting in Lusaka district. Chelstone First Level Hospital is a primary health care facility within the public health care system located about 14km from the Lusaka main post office. At the time of conducting the study, Chelstone First Level Hospital antenatal clinic was offering primary health care through provision of ante-natal care without screening for depression and other common mental disorders to the population of Chelstone and other nearby residential areas every day. A sample size of 281 was calculated using sample size formula for cross-sectional study with a prevalence of 21% from a study in Malawi^25^, precision of 5%, Data for alpha (Za) of 1.96 at 95% confidence intervals. A loss to follow-up (drop out) rate of 10% was considered. The participants were selected through systematic random sampling using a sampling interval of two, recruiting every second antenatal mother into the study after giving consent for participation in the study on the days for antenatal care from October 2019 to December 2019.

The data was collected using a structured socio-demographic and clinical characteristic questionnaire, which was used to capture the demographic, obstetric and other clinical characteristics associated with antenatal depression while depressive symptoms were assessed using The Patient Health Questionnaire-2(PHQ-2) as an initial step and The Edinburgh Postnatal Depression Scale (EPDS), a 10-item screening tool for antepartum and postpartum depression as the second step in the multi-stage assessment for depressive symptoms at Chelstone First Level Hospital as the respondents waited for their routine antenatal assessment. These tools were completed through face-to-face interview by trained Clinical Officer General, Midwife and Clinical Officer Psychiatry, respectively in private consultation rooms. These practitioners were proficient in English and Chichewa to suit the language requirements of the participants. The consistency of their interpretations was checked by a bilingual (English and Chichewa) psychiatry registrar.

The Patient Health Questionnaire-2(PHQ-2) is composed of two questions, each with four possible responses. Each response was assigned a score ranging from zero to three, indicating the severity of a symptom. The total score of a scale ranges from zero to six^26^. A cut- off point of 2 with optimal sensitivity and specificity was determined by undertaking ROC analysis and calculating the area under the curve in SPSS, version 20. Therefore, a pregnant mother with a score of 2 and less was considered to have screened negative for depression while a mother with PHQ-2 score of 2 and more was considered to have screened positive for depression. The internal validity of the instrument was also checked (Cronbach's alpha) which confirms the internal consistency of the tool. The Edinburgh Postnatal Depression Scale (EPDS) is extensively validated and is composed of 10 questions, each with four possible responses. Each response was assigned a score ranging from zero to three, indicating the severity of a symptom. The overall value of a scale ranges from zero (0) to thirty (30). Using a cut-off score of 11/12 on the EPDS for depression, the sensitivity was 88%, and specificity was 87%, with a positive predictive value of 74%, a negative predictive value of 94%, and an area under the curve of 0.8214. The Cronbach's alpha coefficient for the whole scale was 0.87, which ensures the internal consistency of the scale^14^. Consequently, antenatal mothers with a score of 11 and less were normal while mothers with EPDs scoresof 11 and more were depressed. The respondents with a positive test for depression were referred to Chainama Hills College Hospital or University Teaching Hospital Clinic 6 for further assessment and management. A total of 281 pregnant women completed the above questionnaires.

We received ethical clearance from ERES converge Institutional Review Board (IRB) prior to conducting the study and dissemination of results as part of the main project titled, ‘*Validity of the Patient Health Questionnaire-2 as a screening tool for depression among women* attending *antenatal care at Chelstone first level hospital, Lusaka’* (Reference Number 2019-Feb-001). We also got authorization to conduct the study from National Health Research Authority (NHRA), Lusaka District Medical Office and Chelstone First Level Hospital Management before commencement of data collection. The respondents were asked for voluntary participation and written consent was obtained using English and Chichewa through an interpreter on sight after discussing the purpose, benefit, and possible risks of the study. The participants' autonomy was respected throughout the interview. They were made aware about the voluntary nature of the study and their freedom to discontinue at any stage without any consequences. Furthermore, the participants were informed that the interview would provoke some emotions in them and if they got uncomfortable, they were free to withdraw. The benefits of the study such as relief from talking about the problem and referral for help as was deemed necessary were also explained to the participants. Confidentiality was upheld during and after the interview and each participant was assigned a number and assured that their names would not be used in the report. Privacy was assured by conducting the interview in a private consultation room.

The Statistical Package for Social Sciences (SPSS), version 20 was used for data entry and data analysis. Descriptive statistics such as percentages, means, and standard deviation and range were used to summarize the data in tables and charts. Correlation analysis (Pearson Correlation) was carried out between antenatal depression and the predictors/correlates of antenatal depression. Thereafter, a multivariate logistic regression model was generated to identify the independent predictors of antenatal depression to identify potential confounding factors. The dependent variable was antenatal depression while the predictors and independent variables were the sociodemographic and clinical characteristics such as age, marital status, and level of education, monthly income, employment status, and history of medical or obstetric complications, quality of communication with spouse /significant other and unplanned pregnancy.

## Results

### Prevalence of Depressive Symptoms in Pregnant Mothers

The 2-items of the Patient Health Questionnaire-2 (PHQ-2) were aggregated to generate a single variable. The new variable ranges from 0 to 6 in absolute value. Using a cut-off score of 2, a total of 94 (33.5%, 95% CI; 28%, 39%) women screened positive for depressive symptoms. Whereas 187 (66.5%) pregnant women had PHQ-2 scores less than 2, which indicates a negative screening result for depressive symptoms.

We further describe the depression outcomes on the longer screening tool used in the present study, The Edinburgh Postnatal Depression Scale (EPDS) in two levels (depressed and not depressed).

Scores greater than or equal to 11 were used as an indicator for the existence of depression and no depression otherwise. Consequently, about one in four (26.3 %, 95% CI; 21%, 32%) women had symptoms suggestive of antenatal depression.

### Socio-demographic Attributes of Pregnant Women

281 pregnant women were included in the study, encompassing the first, second and third trimesters, with a response rate of 100%. The majority were between the age of 21–29, with a frequency of 149 (53%). Close to two-in-five women attained a secondary education and most 215 (76.5%) of them were married. Approximately three-in-five (61.2%) of the women were unemployed and most (97.9%) women practiced some form of religion. Nearly one in two women (52%) earns less than 100 Kwacha per month (Table 1).

**Table 1:** Socio-demographic Attributes of pregnant women at Chelstone First Level Hospital, Lusaka, 2019

Variables	Values	Frequency (n=281)	Percent (%)
Age (in years)	≤ 20	41	14.6
	21–29	149	53.0
	30–34	63	22.4
	≥35	28	10.0
Marital Status	Single	57	20.3
	Married	215	76.5
	Separated	7	2.5
	Divorced	2	0.7
Level of Education	None	14	5.0
	Primary	83	29.5
	Secondary	117	41.6
	Tertiary	67	23.8
Total Regular Monthly Income (In Kwacha)	<100	146	52.0
	100–500	24	8.5
	600–1000	23	8.2
	1100–1600	23	8.2
	>1700	65	23.1
Employment Status	Employed	109	38.8
	Unemployed	172	61.2
Practice Religion	Yes	275	97.9
	No	6	2.1

### Obstetric Characteristics of Pregnant Women

Three in five pregnant women 169 (60.1%) had a gestation age below 30 weeks. 88 (31.3%) of the respondents had their first pregnancy. Only 35 (12.5%) had more than three pregnancies. Out of 281 pregnant women, 59 (21.0%) had a history of miscarriage whilst 11(3.9%) had a history of stillbirth during labor. Nearly one in three women (30%) reported experiencing an obstetric/medical complication in the index pregnancy (Table 2).

**Table 2:** Obstetric Characteristics of Pregnant Women at Chelstone First Level Hospital, Lusaka, 2019

Variables	Values	Frequency N=281	Percent (%)
Planned Pregnancy	Yes	127	45.2
	No	154	54.8
Number of Pregnancies	1	88	31.3
	2	90	32.0
	3	68	24.2
	>3	35	12.5
Gestation Age (In weeks)	<30	169	60.1
	≥30	112	39.9
Current Obstetric/Medical Complications	Yes	30	10.7
	No	251	89.3
History of Miscarriage	Yes	59	21.0
	No	222	79.0
History of Stillbirth	Yes	11	3.9
	No	270	96.1

### Psychosocial Characteristics of Pregnant Women

Majority of women 254 (90.4%) had very good communication with their partner/ significant other. On the other hand, 252 (89.2%) reported to have been receiving adequate support from their partner and an equivalent proportion were satisfied with their relationship with their partner/significant other. Twenty-three (8.2%) pregnant women reported a history of depression in the past. Moreover, 48 (17.1%) pregnant women reported a positive family history of psychiatry Illness. Thirty-nine (13.9%) of the total respondents had a positive history of substance abuse in the index pregnancy (Table 3).

**Table 3:** Psychosocial Characteristics of the pregnant women at Chelstone First Level Hospital, Lusaka, 2019

Variables	Values	Frequency N=281	Percent (%)
Communication with Partner/Significant Other	Good	254	90.4
	Bad	27	9.6
Relationship Satisfaction	Satisfied	252	89.7
	Unsatisfied	29	10.3
Support from Partner	Yes	252	89.7
	No	29	10.3
History of Depression	Yes	23	8.2
	No	258	91.8
Family History of Psychiatry Illness	Yes	48	17.1
	No	233	89.9
History of Substance Use	Yes	39	13.9
	No	242	86.1

### Factors asso ciated with antenatal depression

Socio-demographic factors, obstetric factors, and psychosocial factors were used to identify statistically significant factors. Among all covariates, employment status, communication with the partner, relationship satisfaction, and support from the partner were found to have p-value less than 0.05 from bi-variant logistic regression (Table 4).

**Table 4:** The relationship of depression with demographic, psychosocial and clinical factors (bivariate analysis) among pregnant women at Chelstone First Level Hospital, Lusaka, 2019

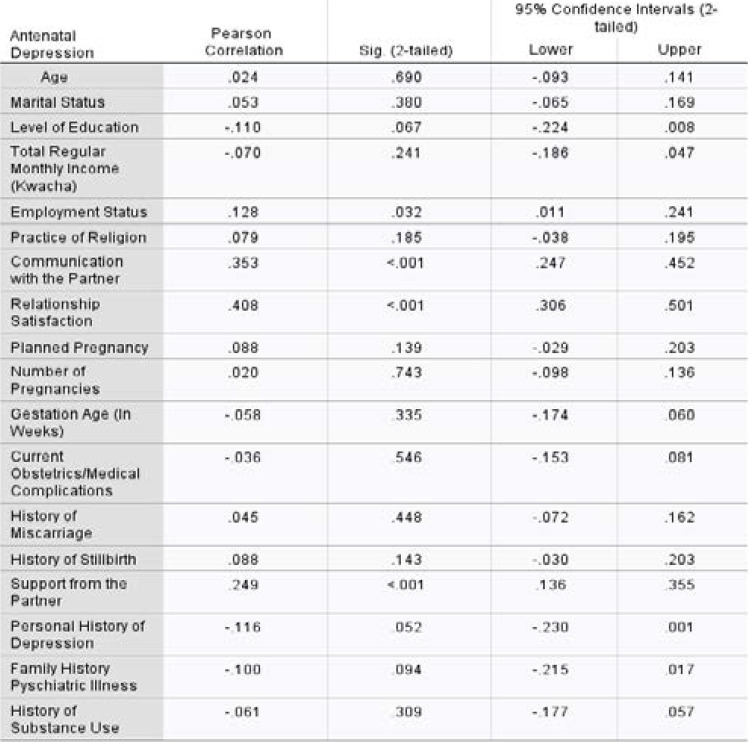

Relationship satisfaction with the partner/ significant other was found to be an independent predictor of antennal depression in the present study. Specifically, the odds of depression increased by 1.7 times (95%CI: 1.4–3.1) for women who were not satisfied with the relationship they had with their partner/significant other (Table 5).

**Table 5:** Factors associated with antenatal depression among women attending antenatal care at Chelstone First Level Hospital, Lusaka, 2019 (multilinear logistic regression)

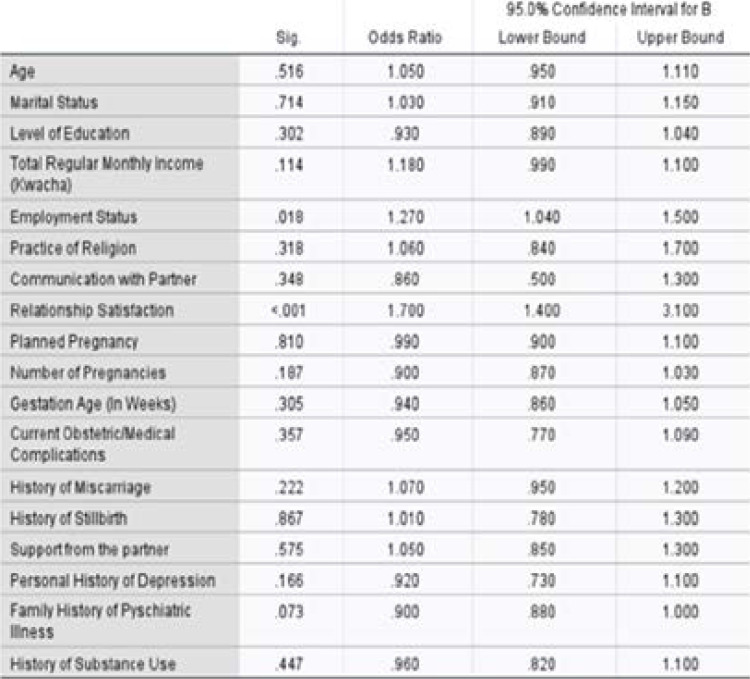

## Discussion

The present study reports that 26.3 % (95% CI; 21%, 32%) of surveyed women had antenatal depression. Our estimate was higher than that reported by a facility-based cross-sectional study conducted among 403 antenatal care attendants in Sodo town of southern Ethiopia (16.3%) using the Edinburgh postnatal depression scale (EPDS)^27^ and another study conducted in Malawi (10.7%) using the Structured Clinical Interview for DSM-IV^25^. Another study conducted in Northeast Ethiopia reported a prevalence rate of 17.9%^28^, which is lower than the findings of the present study. The above differences in prevalence estimates suggest that pregnant women in Zambia may potentially be at an elevated risk of depression compared to pregnant women in Southern Ethiopia and Malawi. The difference in prevalence estimates between the current study and that from southern Ethiopia can be explained by differences in the EPDS cut-off point, whereas the difference between the later study and our study can be explained by differences in depression assessment tools, as each tool has a different ability to discriminate between cases and non-cases. Furthermore, contextual differences may result in different socio-demographic, psychosocial, and clinical characteristics of the respondents in each of the preceding studies and the current study, particularly during this period of severe economic downturn, which poses additional challenges to the pregnant women in the current study and predisposes them to depression.

A study conducted in Zambia among women attending ante-natal care at University Teaching Hospital reported a prevalence rate of 42%, which is higher than that of the present study.^12^ This can be explained by the difference in study setting, in particular the present study was conducted at a primary health care facility while the former was conducted at a tertiary health care facility. This entails different clinical characteristics of the respondents in the two studies, for example the earlier study predominantly had respondents with high risk pregnancies as was noted by the presence of pregnancy induced hypertension and other pre-existing medical conditions thereby increasing the probability of respondents to experience depression as opposed to the respondents of the present study who predominantly had no obstetric and medical complications 251(89.3%) in the index pregnancy.

A cross-sectional study conducted in Northern Uganda among women of mixed Human Immunodeficiency Virus (HIV) status attending ante-natal care at Gulu Regional Referral Hospital found a prevalence rate of 35.8%, which is higher than that of the present study^29^. The prevalence estimates in the present study are also lower than that reported by a community-based, cluster-randomized controlled trial conducted in the outskirts of Cape Town, South Africa (39%)^30^. The above differences can be attributed to the impact of comorbidities. For instance, the majority of the respondents from the earlier study who screened positive for depression had underlying Human Immunodeficiency (HIV) positive status (52.9%), which in itself has been linked to higher rates of depression in pregnancy^31^. Similarly, the study from South Africa reported a higher prevalence rate than the present study due to comorbidities, in particular HIV, tuberculosis, and alcohol use disorder among the respondents.

A systematic review titled, *'Epidemiology of maternal depression, risk factors, and child outcomes in low-income and middle-income countries'* reported a pooled prevalence of 25.8% (95% CI 22·8–29·0%) for depression in pregnancy^32^, which is somewhat consistent with the finding of the present study. The finding of the present study falls within the range of the prevalence rates (11.6%-59%) reported by another systematic review conducted in Ethiopia^33^. This can be explained by the similar study designs and similar World Bank classification of the settings in which the studies included in the systematic review were conducted.

In the present study, antenatal depression was significantly higher among women who had no relationship satisfaction with their partner/significant other. These pregnant women were two times more likely to experience depression compared to those who were satisfied with the relationship they had. This finding is in compliance with the results of previous studies among pregnant women in various countries^34^,^38^ and another study that showed that mental health in pregnancy is significantly affected by social issues like relationship satisfaction, communication within the family dynamic^36^. This association is attributable to the physiological and psychological changes in pregnancy, which influence women to seek out positive partner attributes without which the probability of experiencing depression in the index pregnancy is increased^36^. Moreover, women experience an increase in sexual problems during the early months of pregnancy, which affects the quality of their relationship with the partner and in turn contribute to antenatal depression^28^. In light of the above, prevention efforts for antenatal depression should aim to enhance relationship satisfaction between the pregnant women and their partner^31^,^37^–^38^. This can be done by enhancing positive communication, emotional closeness, instrumental and emotional support, thereby minimizing conflict between partners The implication of this finding for psychiatry in Zambia and other low-middle income countries is that this contributing factor to antenatal depression can be modified from the social perspective through sensitization programs and policy changes like the integration of mental health care into focused antenatal care thereby improving the mental health of pregnant women. Moreover, Brown and Harris addressed the issue of causality in their earlier work on the social origins of depression by providing a female befriender to women who lacked a supportive partner using a randomized controlled trial^39^. This intervention helped vulnerable women cope with life's challenges and prevented them from developing depression. In addition, other researchers have tested an intervention with depressed patients who live with a critical partner^40^. Unlike Brown and Harris' befriending trial, they hired two couple therapists to help the patient improve her relationship with her partner. Couple therapy was found to be more effective and more acceptable than antidepressants in treating depression and preventing relapse. The patient's depression improved due to less hostility from their partner^40^.

This study also found that pregnant women who were not employed were 1.3 (95% CI:1.04–1.5) times more likely to experience antenatal depression compared to those who were employed. This finding is in agreement with earlier studies that found a statistically significant association between depression and unemployment^41^,^42^,^43^. This finding corroborates the findings of the Commission on the Social Determinants of Health that the most socially and economically disadvantaged women have the highest prevalence of common perinatal mental disorders^44^. Gender-based factors such as role restrictions regarding housework and pregnancy care, and excessive unpaid workloads, particularly in multigenerational households with little autonomy for a daughter-in-law, all contribute to risk^42^.

In the present study, it was found that factors like age, level of education, total regular monthly income (in Kwacha), planned pregnancy, practice of religion, gestation age (in Weeks), current obstetric/medical complications, history of miscarriage, history of stillbirth, history of depression and family history of Psychiatry Illness had no significant association with antenatal depression. This finding is contrary to another study where younger age and low levels of education were strong predictors of antenatal depression^45^. The findings of the present study differs from a cross-sectional observational survey conducted at the outpatient department (OPD) of the department of Obstetrics and Gynecology of a tertiary care hospital in Navi Mumbai, which found a significant association of antenatal depression with factors like number of pregnancies, unplanned pregnancy, history of miscarriage and history of obstetric complications^46^, and another cross-sectional study which reported a significant association between antenatal depression and substance use during pregnancy^47^. These variations can be attributed to the differences in the study settings, for example a study done at a tertiary facility may give different contributing factors to antenatal depression when compared to a study done at a primary health care facility. For instance, a study conducted at University Teaching Hospital in Zambia predominantly had pregnant women with high risk pregnancies^12^ while the present study was predominantly conducted among pregnant women with no complications in the index pregnancy-a feature that explains why obstetric complications were not significantly associated with antenatal depression. The present study may not have found an association between antenatal depression and substance use in pregnancy because the majority 242 (86.1%) of the respondents had no history of substance use in pregnancy, a feature that is contrary to an earlier study^47^.

## Strengths and Limitations of the Study

The strengths of this study include recruiting a large sample size, sample diversity, avoiding interviewer bias by using a longer screening tool administered by trained researchers blinded to the results of the initial screening instrument (PHQ-2) and the performance of multivariate logistic regression to remove confounding factors.

Nonetheless, the present study also had some limitations that need to be considered when interpreting the findings of the study. First, the two items in the PHQ-2 do not screen for suicidal ideations, so if the PHQ-2 is used alone, one can miss out respondents presenting with suicidal ideations. This was addressed by using the EPDS which has an item that screens for suicidal thoughts. Second, Participants were drawn from one study site, so the results may differ somewhat in other clinical settings. This limitation was addressed by using simple random sampling of the study site so that results can be generalizable to other populations with similar sociodemographic characteristics in low-middle income countries. Finally, the present study used a cross-sectional design. This was addressed by the large sample size.

## Conclusion

The findings indicate that depressive symptoms are prevalent among pregnant women undergoing antenatal care in primary health care and that unemployment, as well as a lack of relationship satisfaction with a partner or significant other, increased the likelihood of depression. Interventions aimed at increasing relationship satisfaction and empowering pregnant women with employment skills would help prevent antenatal depression. Antenatal care providers should suspect depression in pregnant women who report unemployment and dissatisfaction with their partner/significant other during routine screening and offer targeted interventions to prevent depression's consequences during pregnancy.

## Recommendations

### Policy Recommendations

• Considering that relationship satisfaction between the pregnant woman and the husband/partner/significant other was found to contribute to the likelihood of the pregnant woman being depressed, there is need for the Ministry of religious affairs and national guidance, Churches and Non-Governmental organization to come up with a deliberate policy of introducing couple therapy sessions which will improve harmony between the pregnant woman and husband/partner/significant other thereby reducing the likelihood of depression.

• There is need for The Ministry of Health to emphasize the initiative of male engagement in the care of the pregnant woman so that they are aware of the responsibilities that are forthcoming, the changes in the emotional health (mood) and physical health of their partner. This will help equip the male partner with skills on how to manage the challenges that the woman is likely to face during the pregnancy and cooperate with her at every step of the way so that they are both prepared to receive and manage the child when it arrives.

• Because of the high prevalence of antenatal depression, the Ministry of Health must consider integrating mental health services into primary health care, which will aid in the prevention of complications through early detection and treatment. This will aid in the fight against the stigma associated with mental illness.

• Following the findings of this study there is need for the Mental Health Practitioners and professional bodies like Zambia Psychiatric Association in Zambia to make use of these results as an advocacy tool that can be useful in lobbying for the placement of Mental Health Services as a priority area when it comes to resource allocation thereby improving mental health care in Zambia.

### Recommendations for Further Research

There is a need to carry out the following studies.

• A longitudinal study to assess the causal relationship between antenatal depression and demographic, psychosocial and clinical factors.

• Association of gender disadvantage factors with antenatal depression in women.

• Impact of gender-based violence on antenatal depression.

• Women's views and experiences of having their mental health needs considered in the ante-natal period.

• The impact of partner support on antenatal depressive symptoms.

• The impact of socio-economic status on antenatal depressive symptoms.

## Data Availability

All relevant data for the second and third objectives of the main study are included in the paper.

## Abbreviations

ANC: Antenatal Clinic
CI: Confidence Interval
EPDS: Edinburgh Postnatal Depression Scale (EPDS)
PHQ-2: Patient Health Questionnaire-2
MDD: Major Depressive Disorder
WHO: World Health Organization
HIV: Human Immunodeficiency Virus
